# Nonviral Gene Targeting at rDNA Locus of Human Mesenchymal Stem Cells

**DOI:** 10.1155/2013/135189

**Published:** 2013-05-14

**Authors:** Youjin Hu, Xionghao Liu, Panpan Long, Di Xiao, Jintao Cun, Zhuo Li, Jinfeng Xue, Yong Wu, Sha Luo, Lingqian Wu, Desheng Liang

**Affiliations:** State Key Laboratory of Medical Genetics, Central South University, 110 Xiangya Road, Changsha, Hunan 410078, China

## Abstract

*Background*. Genetic modification, such as the addition of exogenous genes to the MSC genome, is crucial to their use as cellular vehicles. Due to the risks associated with viral vectors such as insertional mutagenesis, the safer nonviral vectors have drawn a great deal of attention. *Methods*. VEGF, bFGF, vitamin C, and insulin-transferrin-selenium-X were supplemented in the MSC culture medium. The cells' proliferation and survival capacity was measured by MTT, determination of the cumulative number of cells, and a colony-forming efficiency assay. The plasmid pHr2-NL was constructed and nucleofected into MSCs. The recombinants were selected using G418 and characterized using PCR and Southern blotting. *Results*. BFGF is critical to MSC growth and it acted synergistically with vitamin C, VEGF, and ITS-X, causing the cells to expand significantly. The neomycin gene was targeted to the rDNA locus of human MSCs using a nonviral human ribosomal targeting vector. The recombinant MSCs retained multipotential differentiation capacity, typical levels of hMSC surface marker expression, and a normal karyotype, and none were tumorigenic in nude mice. *Conclusions*. Exogenous genes can be targeted to the rDNA locus of human MSCs while maintaining the characteristics of MSCs. This is the first nonviral gene targeting of hMSCs.

## 1. Introduction

Human mesenchymal stem cells (hMSCs) are an attractive source of adult stem cells for autologous cell and gene therapy. They have immunosuppressive property and ability to differentiate into multiple cell types present in several tissues [1–3], making them to be a promising cellular vehicle for gene therapy [4, 5]. Current methods most commonly used for genetically modifying hMSCs are based on random transgene integration; however, the uncertainty of the integration site brings problems. Random integration may take place in heterochromatin, leading to silencing [6], or in coding regions, causing disruption of an endogenous gene or interference in the transcription of neighboring sequences [7]. These issues can be addressed by gene targeting, a primary alternative method. Unfortunately, efficient gene targeting in hMSCs has been poorly developed. To our knowledge, only four cases of gene targeting in hMSCs have been reported to date and all of them were based on viral transfer methods. Due to safety concerns related to random integration, nonviral gene targeting at appropriate transgene harbor deserves great attention, while no actual case based on nonviral delivery has been described in hMSCs.

Human cells have approximately 400 copies of a 45S ribosomal DNA (rDNA) repeat that encodes ribosomal RNA (rRNA) and is distributed over the short arm of the five acrocentric chromosomes 13, 14, 15, 21, and 22. The rRNA gene is transcriptionally active and produces approximately 80% of the total RNA in rapidly dividing cells [8]. It is well known that the human rDNA copy number variations are common among healthy individuals, and a balanced chromosomal translocation involving the rRNA cluster occurs without apparent phenotypic effect. The rDNA cluster exhibited strikingly variable lengths between and within human individuals and showed high intrinsic recombinational instability during both meiosis and mitosis [9]. In addition, the human rRNA gene cluster consists of hundreds copies of tandemly repeated rDNA units. Targeting an exogenous gene into one or a few of the rDNA repeats may not cause loss of function effects of the rRNA genes owing to high copy number of this gene. These properties indicate that the rDNA locus may hold a high intrinsic homologous recombination (HR) activity and this locus is considered to be an ideal safe locus for transgene integration [10, 11]. Several studies have reported that efficient targeted gene addition at the rDNA 28s locus could be achieved based on viral transfer methods. However, because the majority of the integration was randomly located in the genome, the risks of the random integration were unavoidable for these methods [10–12]. Alternatively, based on a nonviral vector, in our previous studies, gene targeting at the rDNA 18S locus had been achieved, and the transgene could be stably expressed ectopically in targeted cells [13, 14]. In this study, a nonviral gene-targeting vector was constructed and targeted gene addition in MSCs was performed at the rDNA locus.

In fact, MSCs are a rare population that comprises only approximately 0.001%~0.01% of the total bone marrow mononuclear cells. In addition, gene-targeting frequency in MSCs is intrinsically low. The limited number of the targeted MSCs may be a hurdle for their therapeutic use. Therefore, MSCs genetically modified for clinical application typically require extensive expansion *in vitro*. Unlike embryonic stem cells that have an unlimited proliferative lifespan, adult MSCs *in vitro* display a restricted proliferative longevity, a diminishing replication capacity, and an increased loss of differentiation potential [15–17]. The role of growth factors in enhancing the proliferation and survival of MSCs has been widely studied over the past few years. Several suitable factors have been found to improve *ex vivo* expansion in MSCs without altering their stem cell phenotype and multipotent differentiation potentials, including fibroblast-growth-factor- (FGF-) 2, epidermal growth factor (EGF) [18], and platelet-derived-growth-factor- (PDGF-) BB [19–21]. The combined effects of the factors have appeared quite robust [17, 19–21]. The aim of this study was to target an exogenous gene at the rDNA locus of human MSCs using the nonviral rDNA-targeting vector. The proliferation capacity of the MSCs was improved significantly by adding growth factors. The exogenous neomycin (Neo) gene was targeted at the rDNA locus of MSCs. The recombinant MSCs were compared to control MSCs with respect to phenotype, plasticity, multipotency, karyotype, and tumorigenicity. This is the first report of any nonviral gene targeting of human MSCs and this method may be an optimal approach to MSC-based disease modeling and gene therapy.

## 2. Materials and Methods

### 2.1. Construction of pHr2-NL

The pHr2-NL plasmid contains a long homologous arm (LHA) corresponding to rDNA +937 to ++6523 and a short homologous arm (SHA) corresponding to rDNA +6523 to +7643. It was generated in five steps. Firstly, a fragment homologous to the rDNA +6523 to +7684 region was amplified from human genomic DNA with the primers (5′-AATCGATTTTGATATCTGAGGCAACCCCCTCTCCTCTTGGGC-3′/5′-GTCGCCGCCGGGGACACGCGAA-3′). A fragment homologous to the rDNA +5513 to +6523 was amplified from human genomic DNA with the primers (5′-GCGGAAGGATCATTAACGGAGCCCGGA-3′/5′-ATAATCGATAGAGGAGAGGGGTTGCCTCAGGCC-3′). The PCR products were cloned into pGEMT, resulting in pGEM-T-SHA and pGEM-T-LS, respectively. Secondly, the expression cassette ECMV-IRES-Neo-SV40PolyA was amplified from the pHrneo [14] plasmid with the primers (5′-ATAATCGATATAACTTCGTATAATGTATGCTATACGAAGTTATTCTTAAGGAATTCCCCCTCTCCCT-3′/5′-ATAGATATCATAACTTCGTATAATGTATGCTATACGAAGTTATTAGACGGTCGACCCGTGCGGA-3′). The PCR product (1.8 kb) was cloned into pGEMT, resulting in pGEM-T-INL. Thirdly, the 1.8 kb *ClaI*/*SacI* fragment from pGEM-T-LS was inserted into the *ClaI* and *SacI* sites of pGEM-T-SHA, generating the plasmid pGEM-T-LS-SHA. Next, the *ClaI/EcoRV* fragment from pGEM-T-INL was inserted into the* ClaI* and *EcoRV* sites of pGEM-T-LS-SHA, generating the plasmid pHr1-NL. Finally, the 5.6 kb *MfeI/SacI* fragment from the T-pHr (constructed previously [14], containing the long homologous arm corresponding to rDNA +937 to +6523) and the 5.5 kb *AAT II*/*ClaI* fragment from the pHr1-NL, treated with T4 DNA polymerase (FERMENTS) and fastAP thermo sensitive alkaline phosphatase (FERMENTS), respectively, were ligated to generate pHr2-NL.

### 2.2. Isolation of MSCs from Bone Marrow and Culture Conditions

Informed consent was obtained from all participants according to a protocol approved by the Ethics Committee of State Key Laboratory of Medical Genetics of China (no. 2010-HUMAN-004). Bone marrow was obtained from the iliac bones of two volunteers. MSCs were isolated by using a Histopaque-1077 density gradient (Sigma) as previously described [22, 23]. The cells were cultured in MSC basal medium (L-glucose Dulbecco minimum essential medium (DMEM-L, Sigma-Aldrich, China) supplemented with 10% foetal bovine serum (GIBCO), 100 U of sodium penicillin/mL, and 100 U of streptomycin sulphate/mL). The mononuclear cells were plated at a density of approximately 5 × 10^5^ cells/cm^2^. Symmetrical colonies became visible on days 5 to 7, and the cells were subcultured at a seeding density of 1 × 10^4^ cells/cm^2^. Growth factors including VEGF (5 ng/*μ*L, PEPRO TECH 100–200), bFGF (10 ng/*μ*L, Invitrogen PHG0263), vitamin C (Vc) (50 *μ*g/mL, Sigma A4544), and ITS-X (insulin-transferrin-selenium-X) (100x Invitrogen 51500056) were added [19, 24, 25].

### 2.3. Fluorescence-Activated Cell Sorting (FACS) Analysis

Surface markers CD31, CD44, CD45, CD73, CD90, and CD105 (BD Pharmingen, Hunan, China) were analyzed according to the manufacturer's instructions.

### 2.4. Colony-Forming Unit-Fibroblast (CFU-F) Assay

MSC cells were reseeded at a concentration of 150 cells per 100 mm dish (2.7 cells/cm^2^). After 14 days, cultures were stained with 0.5% crystal violet (Sigma). Colonies less than 2 mm in diameter and faintly stained colonies were ignored. Colony-forming efficiency was expressed as the relative number of colonies generated from the number of cells seeded.

### 2.5. Cell Proliferation Assay

Numbers of cells and cell viability were measured by counting cells on a hemocytometer using the Trypan Blue dye exclusion method. Cells were cultured in 96-well plates (1 × 10^4^ cells/cm^2^). Seventy-two hours later, the number of viable cells was determined using an MTT assay. The plates were analyzed using a microplate reader at 570 nm.

### 2.6. Stable Transfection in MSCs

MSCs were stably transfected with the plasmid pHr2-NL and linearized with *Ahd*I, by nucleofection using the c-17 pulsing program. The DNA/cells ration was 2 *μ*g DNA/5 × 10^5^ cells. The transfected MSCs were plated in 100 mm dishes at a density of 1 × 10^3^ cells/cm^2^. Twenty-four hours after transfection, the medium was replaced with fresh medium. After culture for another 48 hours, 50 *μ*g/mL G418 was added to the culture medium. The medium was refreshed every third day, and the concentrations of G418 on days 3, 6, 9, and 12 were 200 *μ*g/mL, 100 *μ*g/mL, 100 *μ*g/mL, and 15 *μ*g/mL, respectively. Medium without G418 was added on day 14, and the drug-resistant cells were cultured without G418 for another 3 weeks. Individual colonies were picked and expanded, and the genomic DNA was extracted using PCR and Southern blotting to detect recombinants.

### 2.7. PCR Identification of the Site-Integration Colonies

The primer t-up (5′-GTTATCCGCTCACAATTCCACACAACATACGA-3′) and the primer t-re (5′-GGAGGTCGGGGGGACGGGTCCGAGGA-3′) were used. 

### 2.8. Sequencing the PCR Fragment

PCR products were isolated after migration on 0.8% LMP agarose gels, cloned into the pGEM-T vector, and sequenced with primer-T7 (5′-TAATACGACTCACTATAGGG-3′) and primer-SP6 (5′-CATACGATTTAGGTGACACTATAG-3′). 

### 2.9. Southern Blotting

After overnight digestion with restriction enzymes *Pvu II, Nco I, EcoR I,* and *Hind III* (New England Biolabs, Ipswich, MA, USA), 3 *μ*g of genomic DNA per sample was electrophoresed on a 0.8% agarose gel overnight and then transferred to positively charged nylon membranes (Hybond-N+, Amersham, Piscataway, NJ, USA). DNA molecular weight marker III, digoxigenin-labeled DNA (Roche Diagnostics, Indianapolis, IN, USA), and lambda DNA *Hind III* (TaKaRa, Dalian, China) were used as molecular weight markers. The blots were hybridized with DIG-dUTP-labeled probes overnight at 42°C. After incubation with AP-conjugated DIG antibody (Roche Diagnostics, Indianapolis, IN, USA) and appropriate washing, the signals were detected using CDP-Star (Roche Diagnostics, Indianapolis, IN, USA) as a substrate for chemiluminescence. Probes were generated using DIG-High Prime (Roche Diagnostics, Indianapolis, IN, USA), and the templates were generated using PCR amplification from pHr2-NL. The primers used for probe 1 (P1) were 5′-CCCGGAAACCTGGCCCTGTCTT-3′ and 5′-CTTCGCCCAATAGCAGCCAGTC C-3′, and primers for probe 2 (P2) were 5′-AATGGCCGCTTTTCTGGA-3′ and 5′-TGTGATGCTATTGCTTTATTTGTA-3′.

### 2.10. Karyotyping

About 5 × 10^5^ cells from each of the four targeted MSC colonies were treated with 0.08 *μ*g/mL colcemid (Sigma, St. Louis, MO, USA) for 2.5 hours. Then cells were trypsinized, centrifuged, and incubated in 0.075 M KCl for 30 minutes at 37°C. After fixing with Carnoy fixative, metaphase chromosome spreads were prepared using the air drying method. Thirty metaphase spreads were evaluated per colony.

### 2.11. *In Vivo* Implantation Assay

All animal protocols were approved by the Animal Ethics Committee of the State Key Laboratory of Medical Genetics of China. Twenty-four SCID mice were divided into four groups of six mice each. PBS and a total of 2 × 10^6^ cells of each of the three cell types (heterogenous MSCs derived from the four targeted MSC colonies, wild-type MSCs, and HT1080) were injected subcutaneously over the right ribcage. The skin and underlying soft tissue of the relevant area were dissected, fixed in 4% paraformaldehyde, stained with hematoxylin and eosin, and investigated for possible tumor growth.

### 2.12. Differentiation Assays

The four MSC colonies subjected to site-specific integration (1-1, 1-2, 2-1, 2-2) were assessed for adipogenic, osteogenic, and chondrogenic potential. Assays of *in vitro* differentiation to osteocytes, chondrocytes, and adipocytes were performed using StemPro Osteogenesis Differentiation Kit, StemPro Chondrogenesis Differentiation Kit, and a StemPro Adipogenesis Differentiation Kit according to the manufacturer's protocol.

### 2.13. Statistical Analysis

Data sets were expressed as the mean value and standard deviation. The significance of colony-forming efficiency was determined using the Student's *t*-test. The viable cell numbers derived from media with different additives and the number of oil-red-O-positive cells derived from wild-type and targeted MSCs were analyzed using one-way ANOVA. Differences were considered significant at *P* < 0.05.

## 3. Results

### 3.1. Proliferation and Survival of MSCs Treated with Growth Factors

**Figure 1:**
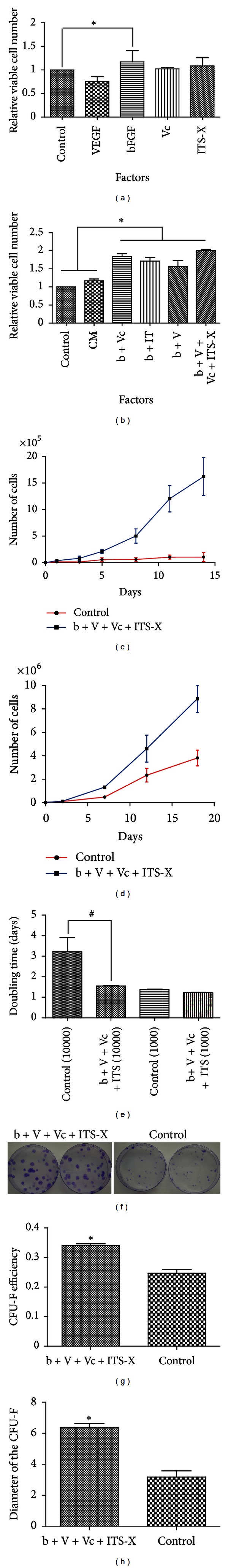
Effects of VEGF, bFGF, VC, and ITS-X on MSC proliferation. The effects of culture conditions with growth factors alone (a) and (b) in combination were examined on viable cell yield as assayed by MTT. MSC growth curves were generated at plating densities of (c) 1 × 10^4^ cells/cm^2^ and (d) 1 × 10^3^ cells/cm^2^. (e) Doubling time was calculated (*n* = 3) at these two plating densities. ^#^ *P* < 0.05. (f) CFU-Fs were stained with crystal violet and captured using a camera (Sony). (g) CFU-Fs efficiency (*n* = 3). (h) The average diameter of CFU-Fs from each set of culture conditions. b, bFGF; V, VEGF; Vc, vitamin C; ITS-X, insulin-transferrin-selenium-X; CM, commercial medium from Stem Cell Technologies; control, DMEM with 10% FBS. *P < 0.05.

First, the effects of several growth factors, including bFGF, VEGF, Vc, and ITS-X were individually evaluated on the proliferation of MSCs. During a 5-day culture period bFGF significantly increased the number of viable cells relative to cells exposed to plain basal medium, but Vc, ITS-X, and VEGF did not (Figure 1(a)). The cumulative numbers of MSCs cultured in the mediums supplemented with bFGF, Vc, VEGF, and ITS-X were 1.47-, 1.05-, 0.78-, and 1.13-fold higher than those in the basal medium. Next, the effects of the basal medium supplemented with various combinations of growth factors on the proliferation of MSCs were evaluated. The results showed that all combinations of growth factors increased proliferation. The cumulative numbers of MSCs cultured in the medium supplemented with Vc+bFGF, VEGF+bFGF, ITS-X+bFGF, and VEGF+bFGF+Vc+ITS-X were 2.06-, 1.77-, 1.63-, and 2.39-fold higher than those in the basal medium (Figure 1(b)). At the plating density of 1 × 10^4^ cells/cm^2^, the cumulative cell numbers from 3 × 10^3^ cells at passage 6 were on average 15-fold (Figure 1(c)) higher than those in the basal medium after 14 days of incubation. They showed a doubling time of about 1.6 days. At a plating density of 1 × 10^3^ cells/cm^2^, the cumulative cell numbers were on average 2.7-fold higher than the basal medium after incubation for 18 days (Figure 1(d)), with a doubling time of about 1.25 days (Figure 1(e)). When the total cell populations were evaluated using a CFU-F assay, the colony-forming efficiency was 34% with the combination of VEGF+bFGF+Vc+ITS-X, which was significantly higher than that in the basal medium (24.7%). The colonies in the VEGF+bFGF+Vc+ITS-X group were clearly larger than the colonies in the basal medium (Figures 1(f)–1(h)).

### 3.2. Gene Targeting of Human MSCs

**Figure 2:**
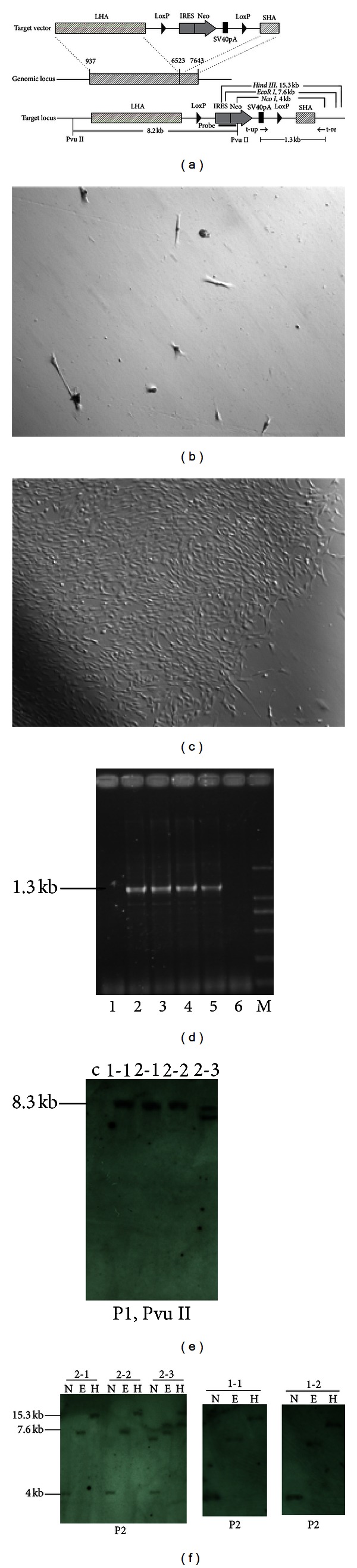
Site-specific integration at the rDNA locus of MSCs. (a) Schematic of the construction of pHr2-NL. pHr2-NL contained two inverted expression cassettes, one consisting of an IRES element from the encephalomyocarditis virus, the coding region of the Neo gene, the SV40 polyA signal (SV40pA), and two loxP sites with the same orientation. LoxP sites were recognized by CRE enzyme to remove the Neo cassette after gene targeting. LHA, long homologous arm (U13369:937-6523); SHA, short homologous arm (U13369:6523–7643). The genomic locus indicates the 6.7 kb fragment (U13369:937-7643) required for homologous recombination at the internal transcribed spacer 1 (ITS1) of the rRNA gene. Single cutting sites for restricted enzymes of *Nco I, EcoR I, Hind III*, and *Pvu II * are located at the IRES-Neo frame and outside of the long homologous fragment. The fragment between the two *Pvu II* sites was 8285 bp in size, and it was detected using probe 1 (P1). The expected sizes of the restriction fragments produced by *Nco I, EcoR I, and Hind III * were 4001 bp, 7628 bp, and 15,316 bp, respectively. These were detected using probe 2 (P2). Primer t-up was located at the SV40 polyA. Primer t-re was located outside of the SHA at the hrDNA locus. (b) Drug-resistant cell in basal medium. (c) Drug-resistant colonies in the medium supplemented with VEGF+bFGF+Vc+ITS-X. (d) Identification of colonies with site-specific integration by PCR. The expected fragment, 1.3 kb in size, was amplified from the genomic DNA of colonies using site-specific integration. M, DL200 DNA marker; 1, negative colony; 2–5, positive colonies; 6, wild-type MSCs. (e–f) Southern blotting analysis of the representative recombinants. Genomic DNA digested with *Pvu II*, *Nco I, EcoR I, *and *Hind III* was analyzed. A specific band was consistently detected in colonies 1-1, 1-2, 2-1, and 2-2. An additional band beside the specific band was detected in colony 2-3. c, control (untransfected MSCs); *N, Nco I; E, EcoR I; H, Hind III*.

We constructed an rDNA-targeting plasmid, pHr2-NL, which introduced a promoterless neomycin (Neo) cassette flanked by two loxP sites into the 45S pre-RNA gene. The cassette was flanked by a long homologous arm (5.6 kb) and a short homologous arm (1.1 kb). The cassette contained an encephalomyocarditis virus internal ribosomal entry site (EMCV-IRES), which enabled resistant gene expression under the control of endogenous RNA polymerase I (Pol I) promoter upstream after HR (Figure 2(a)).

A targeting experiment was first carried out in HT1080 cells. The enrichment efficiency was 50% and the targeting efficiency was 0.01% (Table 1). Then the targeting experiment was performed in triplicate on two groups of MSCs. In the groups exposed to basal medium, a few of drug-resistant cells can be observed but there were no colonies (Figure 2(b)). When exposed to VEGF+bFGF+Vc+ITS-X, many tight colonies were observed (Figure 2(c)). PCR was initially used to detect the positive recombinants; 2 out of 9, 3 out of 23, and 11 out of 50 drug-resistant colonies were found to contain positive recombinants (Table 2) (Figure 2(d)). PCR-positive recombinants were detected by Southern blotting after 5 passages; the results showed only one 8.3 kb band, which indicates that the site-specific integration of the exogenous cassette at the rDNA locus without random integration was present in 4 out of 5 representative PCR-positive colonies. However, an unexpected extra band appeared in one of the PCR-positive colonies, indicating that random integration also took place (Figure 2(e)). Consistent results were produced when the genomic DNA was cut with* Nco I, EcoR I,* and *Hind III* (Figure 2(f)).

**Figure 3:**
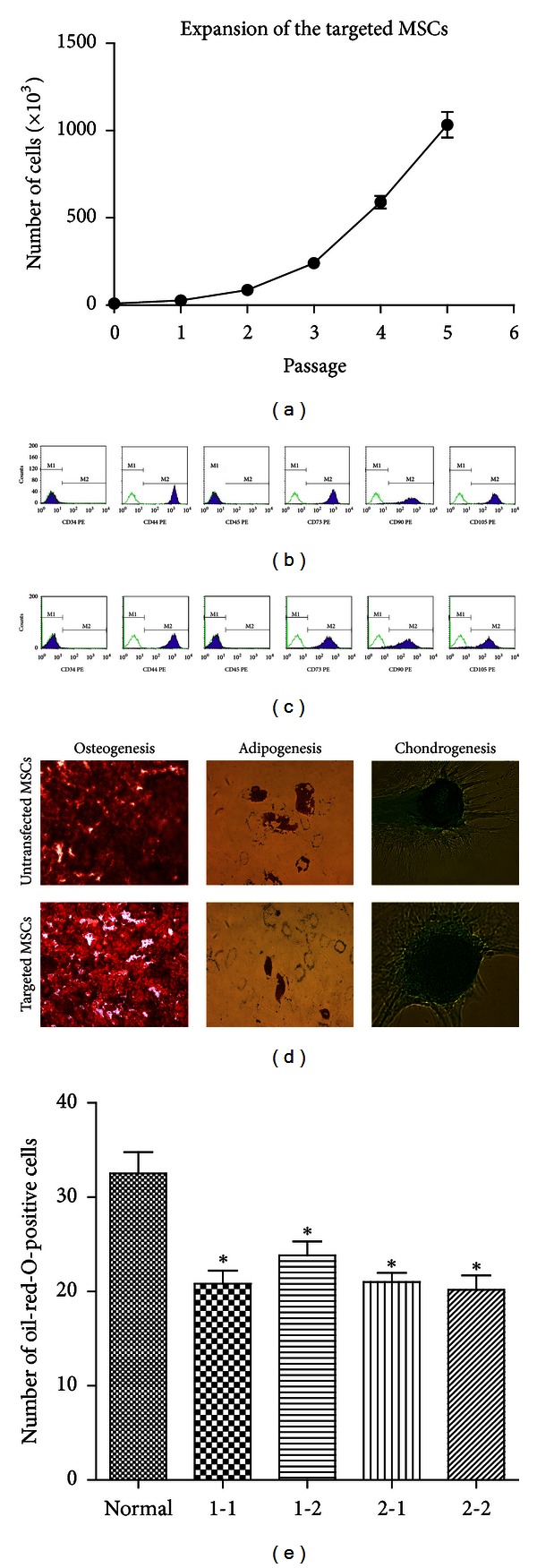
MSC surface antigen and differentiation potential detection. (a) Expansion of the targeted MSCs (*n* = 3). About 1 × 10^4^ cells from every targeted colony were expanded. Cell count was performed at every passage. The expanded MSCs were subjected to MSC surface antigen and differentiation potential detection. (b) Flow cytometry analysis of the surface antigen expression of hMSCs untransfected. Green curves represent isotype controls and blue curves represent the specific antibodies. (c) Flow cytometry analysis of the surface antigen of expanded MSCs with gene targeting. (d) Adipogenic, osteogenic, and chondrogenic potential of hMSCs untransfected (upper) or with gene targeting (down). The adipogenic cultures were stained with oil red O to measure the accumulation of intracellular lipids. The osteogenic cultures were stained with alizarin red S to detect calcium deposition. For chondrogenic induction, the pellet sections were stained with alcian blue dye to detect proteoglycans. (e) Quantitative analysis of the adipogenic differentiation. Normal MSCs at passage 6 (normal) and targeted MSCs (1-1, 1-2, 2-1, 2-2) were differentiated into adipocytes. For each colony, the number of the oil-Red-O-positive cells was counted under 6 microscope fields. *P < 0.05. The bar indicates 50 *μ*m.

**Figure 4:**
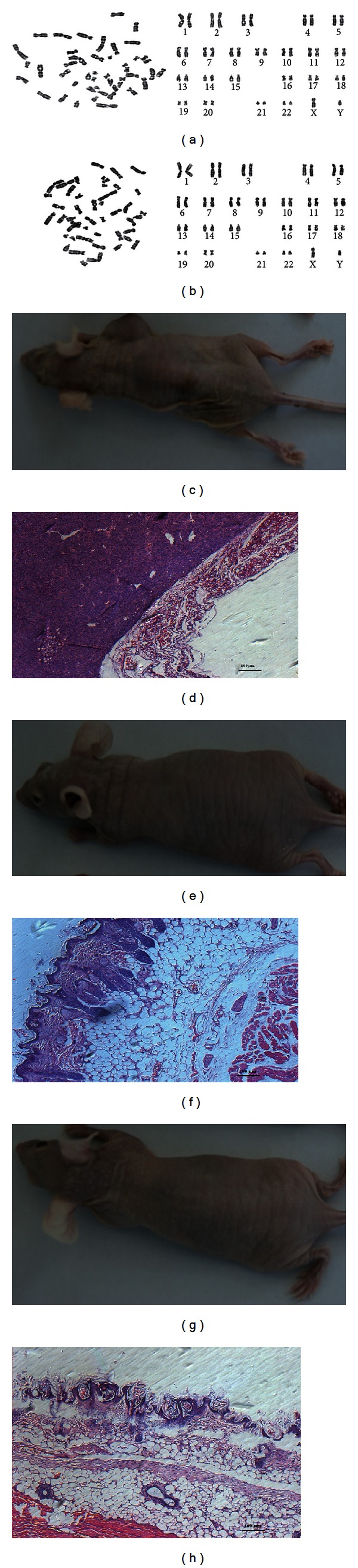
Karyotyping and *in vivo* tumor formation. Karyotyping revealed that the (a) untransfected MSCs and (b) MSCs subjected to gene targeting maintained a normal karyotype: 46, xy. (c–h) *In vivo* tumor formation. Untransfected MSCs and those targeted with an exogenous gene did not show (e, g) macroscopic or (f, h) microscopic staining after 8 weeks. (c) In contrast, HT1080 formed macroscopically visible tumors within 4 weeks. (d) Hematoxylin and eosin staining revealed a characteristic tumor growth. The bar indicates 50 *μ*m.

The MSC colonies that underwent gene targeting were expanded and the cell numbers were counted. On average, 1 × 10^7^ cells were obtained from one targeted MSC colony (Figure 3(a)). The expanded MSCs retained the MSC surface antigene expression (Figures 3(b) and 3(c)) and the ability to differentiate into chondrocytes, adipocytes, and osteocytes *in vitro* (Figure 3(d)). Quantitative analysis indicated that the adipogenic differentiation partially decreased compared with the normal MSCs at passage 6 (Figure 3(e)). They retained a normal karyotype (Figures 4(a) and 4(b)) and failed to develop tumors *in vivo* (Figures 4(c)–4(h)).

## 4. Discussion

Recent advances have shown that the use of MSCs as therapeutic vehicles may be feasible. The development of gene-targeting methods based on nonviral transfer for hMSCs deserves attention with respect to the advantages of nonviral vectors. The advantages of nonviral gene transfer include low acute toxicity, simplicity, few restrictions on the size of the gene of interest, and feasibility to be produced on a large scale [26–29]. Here, we established a nonviral method and demonstrated that the exogenous Neo gene could be targeted to the rDNA locus of MSCs. The gene-targeted MSCs maintained uniform surface antigen expression and a normal karyotype and did not develop tumors *in vivo*. Nonviral methods based on transposons such as Sleeping Beauty and piggyBac have been reported to be efficient in gene therapy and to be comparable in time-consuming compared with this method. However, safety issues about the transposons are reported. The first safety issue is about the presence of the SB transposase gene and the potential for remobilization of transposons already sited in the recipient genome. The second one is the insertional mutagenesis. The Sleeping Beauty transposon has the most random integration preference of the vectors currently in use for gene therapy [30, 31]. In this study, following antibiotic selection using G418 based on a promoter-strap strategy, the site-specific integration recombinants were selected by PCR and Southern blot assays and expanded *in vitro*. The random recombinants were eliminated.

The low integration efficiency of nonviral gene-targeting addition in mammalian cells has been a major limitation to its application [32]. It is thought that nonhomologous end joining (NHEJ) and HR DNA-repair pathways mediate random integration and site-specific integration, respectively. NHEJ is believed to occur at rates that are three to four orders of magnitude higher than those of HR [33], which makes it relatively easier to obtain colonies that carry a randomly integrated transgene. In this study, the relative gene-targeting frequencies achieved in the rDNA locus were observed to be 13%–22% in hMSCs and 50% in HT1080 cells. By using the nonviral delivery method, the absolute targeting frequency is more than 20-fold higher than that in HT1080 cells at *HPRT*, a most commonly targeted locus [34]. In previous reports, by including 28S rDNA homology arms into the vector design, the integration frequency of a recombinant adeno-associated viral vector in rat hepatocytes was enhanced by 30-fold [10]. The underlying mechanism appears to be the relatively high intrinsic activity of HR at the rDNA locus. Studies on Arabidopsis thaliana and yeast cells have suggested that the rDNA region may have functional components that stimulate HR [35]. Based on the similarity of rDNA structures between different eukaryotic cells, the rDNA region may be a common HR hotspot in the majority of eukaryotic cells [36]. Despite recent success of gene targeting mediated by zinc-finger nuclease (ZFN) [4], we chose not to pursue this strategy because of the laborious design process and the toxicity resulted from the “off-target” effects [37].

To obtain enough cell for clinical use, the MSCs modified at a low efficiency need extensive expansion *in vitro*. Because the gene-targeting efficiency of nonviral vectors was relatively low, there exists a need to expand the targeted cells to get the requisite cell number. Previous reports have shown that by supplementing growth factors the culture conditions could be optimized for inducing proliferation of MSC while maintaining their multipotency. Although combination of two or more supplements have been used [20, 21], the synergistical effects of the factors on the proliferation of MSCs were rarely reported [19]. Our results show that bFGF is critical to MSC growth and that it acted synergistically with vitamin C, VEGF, and ITS-X, causing the cells to expand significantly. Masahiro found continuous FGF stimulation to be necessary for the maintenance of VEGFR2 levels in mice modulating sensitivity to VEGF stimulation [38]. This may explain the synergistic effects of VEGF and bFGF on the proliferation of MSCs. Although the details of the mechanism by which VEGF, bFGF, Vc, and ITS-X synergistically increase cell proliferation are unclear, the most robust growth stimulation was observed with VEGF+bFGF+Vc+ITS-X (Figures 1(c) and 1(d)). In the medium without supplements, after antibiotic selection, for example, using G418, a few of the resistant MSCs could be observed but they did not form large cell clones and even did not expand to sufficient numbers for characterization (Figure 2(b)). By adding the supplements to the culture medium, the proliferation capacity of the MSCs was obviously improved and at least 1 × 10^7^ cells could be obtained from one targeted recombinant colony. As 11 targeted colonies can be obtained from 3 × 10^6^ MSCs transfected, the total cell number could be calculated as 1.1 × 10^8^. The amount of this level could meet requirement of clinical use (10^7^~10^8^) [39]. The expanded MSCs retained multipotency, although the adipogenic differentiation partially decreased.

In addition, a second cell behavior critical for the successful use of MSCs is the survival of the cultured cells. The increased survival of MSCs stimulated by growth factors, such as VEGF [25] and Vc [40], may help single targeted MSCs to form colonies. Transferrin and selenite can reduce toxic levels of oxygen radicals and be used as antioxidant in the medium [41]. During the selection process by G418, untargeted MSCs killed may release cytokines to the culture medium. This may increase the survival stress of the targeted MSCs. It was reported that high survival stress such as the oxidative stress could promote cell senescence [42]. This may be why the untargeted MSCs could form colonies but the targeted MSCs cannot form colonies in the basal medium without growth factors. Further improvements proliferation and survival of MSCs by formulation optimization of the different additives and improvement targeting efficiency by optimization of the targeting conditions may help to get more MSCs with targeted modification.

In summary, this study is the first to describe gene targeting of hMSCs using a nonviral delivery system. Exogenous therapeutic genes could be targeted to the rDNA locus of MSCs using the hrDNA vector described herein, and desirable number of the targeted cell could be obtained by improving the proliferation capacity of the MSCs using growth factors. This shows that MSCs have potential as a cellular vehicle for clinical use, and we believe that this method may be useful for autologous therapy of monogenic inheritance disease. Based on the fact that hFVIII integrated at the rDNA locus of several human cell lines expressed efficiently [14] and MSCs can home to sites of ongoing injury/inflammation to release FVIII [43], hFVIII-expressing MSCs generated using the method described herein may bring great hope for the autologous therapy of the hemophilia A, which is the most common inheritable deficiency of coagulation.

## Figures and Tables

**Table 1:** Gene targeting in HT1080 cells.

Exp.	*N *	*C *	*T *	*S *	ATF	RTE
1	1.0	210	6	12	105	50.0%
2	3.0	529	16	30	94	53.3%

Exp.: experiment performed. *N*: number of cells nucleofected (×10^6^). *C*: total number of resistant colonies obtained from each experiment. *S*: number of colonies screened. *T*: number of colonies screened as targeted recombinants. ATF: absolute targeting frequency (×10^−6^) = *TC*/*NS*. RTE: relative targeting efficiency = *T*/*S*.

**Table 2:** Gene targeting in MSCs.

Don	*N *	*C *	*S *	*T *	ATF	RTE
1#	0.5	17	9	2	7.6	22.2%
2#	1.0	36	23	3	4.7	13.0%
2#	3.0	98	50	11	7.2	22.0%

Don: donor of bone marrow. *N*: number of cells nucleofected (×10^6^). *C*: total number of resistant colonies obtained from each experiment. *S*: number of colonies screened. *T*: number of colonies screened as targeted recombinants. ATF: absolute targeting frequency (×10^−6^) = *TC*/*NS*. RTE: relative targeting efficiency = *T*/*S*. 1#, 2#: bone marrow donors.
